# Comment on “Cerebrospinal fluid camk2a levels at baseline predict long-term progression in multiple sclerosis. Clinical Proteomics”

**DOI:** 10.1186/s12014-023-09433-w

**Published:** 2023-10-25

**Authors:** Fereshteh Behdarvand Dehkordi, Mohammad Chehelgerdi

**Affiliations:** 1 Novin Genome (NG) Lab, Research and Development Center for Biotechnology, Shahrekord, Iran; 2 Young Researchers and Elite Club, Shahrekord Branch, Islamic Azad University, Shahrekord, Iran

Dear Editor,

I am writing to provide a comparative analysis of the recently published study titled “Cerebrospinal fluid CAMK2A levels at baseline predict long-term progression in multiple sclerosis” conducted by Sohaei et al. [1]. (Clinical Proteomics. 2023 Dec;20(1):1–2). Given the significant findings of this study, it is crucial to assess its contributions in relation to existing research to offer a comprehensive perspective on the subject matter.

To conduct a comparative analysis of the study titled “Cerebrospinal fluid CAMK2A levels at baseline predict long-term progression in multiple sclerosis” with other references, we would need to gather related studies that investigate CSF biomarkers in multiple sclerosis. Some points to consider for the comparative analysis could include:

Firstly, future work based on this study should look at expanding the sample size so that results are more generalizable across the MS patient population. This can potentially provide valuable insight into treatment modalities and outcomes. Additionally, the research could expand to consider the interaction of these biomarkers with other predictive or risk factors for MS. This comprehensive approach may contribute to the development of a more robust prognostic tool. Furthermore, a rigorous validation of these biomarkers should be performed in larger cohorts and various stages of disease progression. Moreover, validation of current findings might also reveal whether the predictive power of these biomarkers changes over the course of the disease [2].

In addition, while CAMK2A is a promising biomarker for future exploration, the functional role of this protein in MS pathophysiology should be analyzed in the context of other potential interacting proteins or pathways that could influence disease severity. Lastly, the cost and feasibility of using such an analytical methodology, like targeted liquid-chromatography tandem mass spectrometry, in a clinical setting is another aspect to be considered in future studies. The translation of these findings into actual clinical application may also face significant technological or procedural barriers that will need to be addressed [3].

Given these findings, future work could involve validation of novel CSF biomarkers with a focus on CAMK2A, in additional large-scale, independent cohorts of MS patients, as the published results were obtained from a relatively limited sample size. Further examination could also review these biomarkers’ predictive value in secondary progressive MS, as well as their association with different disease-modifying treatments. Moreover, a longitudinal study design could offer a deeper understanding of their role and variability over time and alongside disease progression. Applying other highly sensitive and specific analytical techniques could also unearth more novel biomarkers, and the integration of these proteomic data with other “omics” data warrants investigation [3, 4].

## Data Availability

Not applicable.
